# Women and stroke: disparities in clinical presentation, severity, and short- and long-term outcomes

**DOI:** 10.3389/fneur.2023.1147858

**Published:** 2023-05-15

**Authors:** Hiba Naveed, Muna Almasri, Bahram Kazani, Areej Nauman, Naveed Akhtar, Rajvir Singh, Saadat Kamran, Salman Al Jerdi, Sathvika Thermalingem, Ashfaq Shuaib

**Affiliations:** ^1^Weill Cornell College of Medicine, Doha, Qatar; ^2^The Neuroscience Institute, Hamad Medical Corporation, Doha, Qatar; ^3^Neurology Division, Department of Medicine, University of Alberta, Edmonton, AB, Canada

**Keywords:** ischemic stroke, women, disparities, outcome, major adverse cardiovascular events

## Abstract

**Objectives:**

There are limited data from the Middle East on sex-related differences in short- and long-term stroke outcomes. We present 8 years of experience based on the Qatar stroke database.

**Setting:**

The Qatar stroke database prospectively collects data on all stroke patients admitted to Hamad General Hospital. For this study, we compared female and male acute ischemic stroke patients on their characteristics at admission, short-term outcomes [modified Rankin Scale (mRS) score], and long-term outcomes [incidence of major adverse cardiovascular events (MACEs)].

**Participants:**

A total of 7,300 patients [F: 1,406 (19.3%), M: 5,894 (80.7%); mean age 55.1 ± 13.3 (F: 61.6 ± 15.1, M: 53.5 ± 12.3; *p* < 0.001)] were admitted with acute ischemic stroke.

**Results:**

Significantly fewer women presented within 4.5 h of onset (F: 29% vs. M: 32.8%; *p* = 0.01). Although women were more likely to experience severe stroke (NIHSS >10; F: 19.9% vs. M: 14.5%; *p* < 0.001), fewer were treated with thrombolysis (F: 9.8% vs. M: 12.1%; *p* = 0.02). Women experienced more medical complications (F: 11.7% vs. M: 7.4%; *p* < 0.001) and tended to have a more prolonged length of stay in the hospital (F: 6.4 ± 7.6 days vs. M: 5.5 ± 6.8 days; *p* < 0.001).

**Primary and secondary outcome measures:**

Good outcomes at 90 days (mRS score of 0–2) were less frequent in women (F: 53.3% vs. M: 71.2%; *p* < 0.001). Fewer female patients were taking antiplatelets (F: 78% vs. M: 84.8%; *p* < 0.001) or statins (F: 81.2% vs. M: 85.7%; *p* < 0.001). Significantly more female patients experienced a MACE (F: 12.6% vs. M: 6.5%; *p* < 0.001).

**Conclusion:**

Older age at presentation contributes to poor outcomes following acute stroke in women. Other contributing factors include delays in admission to the hospital, lower rates of thrombolysis, and lower rates of provision of preventative treatments.

## Highlights

- The strengths of this study include the large sample size and minimal loss to follow-up. Moreover, there are few studies on the long-term risk of major adverse cardiovascular events (MACEs) in women.
- The analytical approach employed was able to significantly reduce residual confounding.
- The single-center nature of the study setting may reduce the generalizability of the findings.
- The data were collected prospectively for entry in the Qatar stroke database, but the analysis was retrospective and therefore did not have prespecified objectives; this might have resulted in low accuracy of the comorbidity profile.
- We may have missed clinical events occurring in patients who did not go to the hospital or who may have received treatment outside the country.

## Introduction

Outcomes following acute stroke may vary depending on the gender of the patient (1, 2). Stroke is more common in women, among whom it is the fourth leading cause of death, as compared to the fifth leading cause in men (3, 4). The death rate following stroke is higher in women (women: 6.2%; men: 4.4%), resulting in 55,000 more excess deaths among women annually (3, 4). Stroke rates are especially high in women in the older age category (>80 years of age) (5). In women, stroke is the most common presentation of cardiovascular disease, whereas the leading manifestation in men is coronary artery disease (6). There is evidence that modifiable risk factors for stroke may affect men and women differently. For example, the effect of diabetes on stroke risk is greater in women compared to men (7). This is especially evident in the case of type 1 diabetes (7–9). Similarly, recent evidence suggests that hypertension may have a stronger association with stroke in women compared to men (8, 10, 11). There is also an association between increased body mass index (BMI), or obesity, and stroke, and this association also appears to be more prominent in women (8). Atrial fibrillation, an important risk factor for stroke, is more frequent in women (7), and the risk of stroke is particularly high in women older than 65 years of age (12). Additionally, there are several important female-specific risk factors that also contribute to the risk of stroke in women (13–15).

Women are less likely to receive appropriate treatment for acute stroke (1, 2). Several factors may contribute to the provision of suboptimal treatment for women. First, the diagnosis of acute stroke is often missed in women. In a recent cross-sectional analysis of misdiagnosis of acute stroke, women had 25% lower odds of the correct diagnosis being made (16, 17). A higher frequency of atypical stroke symptoms in women may contribute to the misdiagnosis of stroke (18). We have previously shown that stroke mimics are more common in women (19). This may also contribute to higher rates of misdiagnosis and delays in treatment. A recent meta-analysis showed that thrombolysis was offered less frequently to women, and that results indicating lower rates of treatment with rt-PA occurred most frequently in reports from Europe and the USA (20). Women are also more likely to have severe symptoms, another factor that contributes to poor outcomes following acute stroke (21).

Most of the sex- and gender-related research on stroke diagnosis, treatment, and outcomes has been published in Europe and North America (1, 2). The effects of gender and sex on outcomes of stroke have not been thoroughly studied in populations in the Middle East or Southeast Asia. Regarding Qatar specifically, we have previously published work on the topics of ethnic variation (22), stroke risk factors (23), clinical presentation (24), thrombolysis in acute stroke management (25), and short-term (26) and long-term (27) outcomes of stroke in the population of Qatar. We have also shown that post-stroke depression tends to be more common in women in Qatar (28). We now present our experience based on analysis of a large database of prospectively collected data on 7,300 patients; using these data, we studied the risk factors, presenting symptoms, administration of reperfusion treatment, course of disease during hospital stay, and short- and long-term prognosis in male and female patients admitted to the hospital following acute stroke.

## Methods

All patients with acute ischemic stroke (AIS) who were admitted to the Hamad General Hospital (HGH) between January 2014 and January 2022 were included in the study. Clinical information was entered prospectively in a database, as reported in previously published work (22–24). The hospital has a dedicated stroke ward, admits more than 95% of stroke patients in Qatar, and is the only center where AIS treatment with intravenous thrombolysis and mechanical thrombectomy is offered. The clinical information collected on all patients admitted to the HGH included prehospital Modified Rankin Scale (mRS) score, vascular risk factors, mode of transportation, symptom severity, time to CT, and “door-to-needle” time in cases where thrombolysis was offered. Clinical evaluation included the severity of stroke as measured by the National Institute of Health Stroke Score (NIHSS). Treatment at admission and discharge was recorded for all patients. We also documented any medical complications that developed during admission, including aspiration pneumonia, bedsores, bladder infections, and any systemic sepsis. All patients were assessed on the mRS at discharge and at 90 days following acute stroke. In the current study, we also reviewed the state-wide Cerner medical records of the patients to document any vascular complications following discharge.

We evaluated differences between men and women in terms of mode of transportation to the hospital, time from onset to presentation to the emergency department, severity of stroke symptoms, type of stroke, and whether the patient was treated with thrombolysis and thrombectomy. In addition, we monitored the rate of complications, length of stay in the hospital, and changes in treatment of risk factors at the time of discharge. The primary short-term outcome measure in the present study was a comparison of mRS scores at 90 days between female and male patients. An mRS score of 0–2 was considered a good outcome, and an mRS score of 3–6 was defined as a poor outcome. For analysis of long-term outcome, we compared the rate of major adverse cardiovascular events (MACEs) among men and women at 1 year. The occurrence of MACEs (defined as cardiovascular mortality; all-cause mortality; fatal or non-fatal myocardial infarction; recurrent stroke; and revascularization procedures, i.e., coronary artery bypass graft or percutaneous coronary intervention) during follow-up after the index event was also compared.

The study was approved by the Institutional Review Board, Hamad Medical Corporation at the Medical Research Center (MRC-01-20-1135) and the Institutional Review Board of Weill Cornell Medicine—Qatar (1932095-1/22-00016).

Informed consent: This was a registry study; hence, consent was not applicable.

### Patient and public involvement

Neither patients nor the public were involved in the design, conduct, reporting, or planned dissemination of our research.

### Statistical methods

Descriptive statistics were used to characterize the study sample. Descriptive results (including graphical illustrations) for all quantitative variables (e.g., age) are presented in the form: mean, standard deviation (SD) for all interval variables; frequencies with percentages are reported for categorical variables. Inferential statistics, such as the results of Student's *t*-tests, were used to evaluate the statistical significance of mean differences between female and male ischemic stroke patients for all interval variables; chi-square tests were conducted to examine the association between sex and categorical variables among ischemic stroke patients. A multivariate binomial logistic regression analysis was conducted to examine the factors that were more common among women in comparison to men after adjusting for significant independent variables, such as age, NIHSS score, systolic blood pressure, diabetes, HTN, prior stroke, smoking, obesity, length of stay, poor outcome at 90 days, and etiology of stroke (TOAST classification). This analysis was conducted using the equation logit *E*(*Y*) = *b*_0_ + *b*_1_*x*_1_ + *b*_2_*x*_2_ + ……+ *b*_n_*x*_n_, where *Y* is the Bernoulli distributed outcome variable of sex (male = 0 and female = 1), *x*_i_ (*i* = 1 to *n*) represents the independent variables, and *b*_i_ (*i* = 1 to *n*) represents the linear parameters. A forest plot based on the same multivariate regression analysis was constructed to illustrate significant predictors of sex. A *p*-value of 0.05 (two-tailed) was considered to represent the threshold for statistical significance. The analysis was conducted using the SPSS 28.0 statistical software package.

## Results

### Demographics

**Figure 1:**
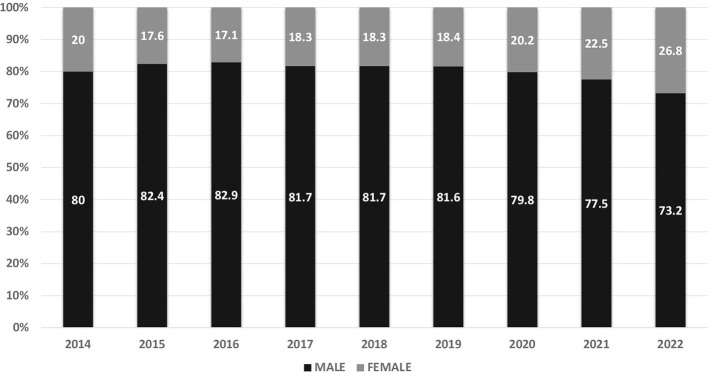
Proportion of male vs. female patients admitted annually in each year during the study.

During the study period (January 2014 to January 2022), 14,768 patients were admitted to the HGH stroke program. The proportions of men and women among the patients admitted in each year are shown in Figure 1. After exclusion of 1,549 patients with ICH, 1,413 patients with TIAs, 204 patients with cerebral venous thrombosis, and 4,302 patients with stroke mimics, data from 7,300 patients with a final diagnosis of acute ischemic stroke [women: 1,406 (19.3%), men: 5,894 (80.7%); mean age: 55.1 ± 13.3 (women: 61.6 ± 15.1, men: 53.5 ± 12.3; *p* < 0.001)] were available for analysis. The larger number of male patients reflects the demographics in Qatar, where there is a predominance of young expatriate male workers. Most women are either local Qatari nationals or long-stay expatriates. As shown in Table 1, hypertension, diabetes, obesity, atrial fibrillation, and prior stroke were significantly more common in women, while smoking was more common in men. Levels of triglycerides and LDL cholesterol at admission were significantly higher in men. Women were significantly more likely to be taking antithrombotic, anti-hypertensive, anti-diabetes, and statin medications. This is likely because of the older average age and higher rates of vascular risk factors among women. While atrial fibrillation may have contributed to the occurrence of stroke, < 50% of female patients were taking anticoagulants at the time of stroke. The data showed that a larger proportion of female patients were in the age group older than 65 years compared to male patients (*p* = 0.001; Table 1).

**Table 1:** Baseline characteristics and risk factors in the female and male population of ischemic stroke patients.

**Variable**	**Overall (*n* = 7,300)**	**Women (*n* = 1,406, 19.3%)**	**Men (*n* = 5,894, 80.7%)**	***P*-value**
Age (mean, years)	55.1 ± 13.3	61.6 ± 15.1	53.5 ± 12.3	< 0.001
**Age category**				
< 46 years	1,807 (24.8)	223 (15.9)	1,584 (26.9)	< 0.001
46–55 years	2,104 (28.8)	229 (16.3)	1,875 (31.8)	
56–65 years	1,863 (25.5)	354 (25.2)	1,509 (25.6)	
>65 years	1,526 (20.9)	600 (42.7)	926 (15.7)	
Hypertension	5,324 (72.9)	1,074 (76.4)	4,250 (72.1)	< 0.001
Diabetes	4,133 (56.6)	880 (62.6)	3,253 (55.2)	< 0.001
Dyslipidemia	3,500 (47.9)	705 (50.1)	2,795 (47.4)	0.07
Prior stroke	872 (11.9)	227 (16.1)	645 (10.9)	< 0.001
Atrial fibrillation on admission	613 (8.4)	213 (15.1)	400 (6.8)	< 0.001
Coronary artery disease	890 (12.2)	173 (12.3)	717 (12.2)	0.89
Active smoking	2,074 (28.4)	49 (3.5)	2,025 (34.4)	< 0.001
Obesity (body mass index ≥30 kg/m^2^)	1,848 (25.3)	577 (41.0)	1,271 (21.6)	< 0.001
Prior antiplatelet use	1,523 (20.9)	405 (28.8)	1,118 (19.0)	< 0.001
Prior anticoagulant use	359 (4.9)	106 (7.5)	253 (4.3)	< 0.001
Prior anti-hypertensive use	2,394 (32.8)	685 (48.7)	1,709 (29.0)	< 0.001
Anti-diabetic medication use	1,882 (25.8)	503 (35.8)	1,379 (23.4)	< 0.001
Prior statin use	1,629 (22.3)	467 (33.2)	1,162 (19.7)	< 0.001
RBS on admission (mmol/L)	9.6 ± 4.9	9.9 ± 5.0	9.5 ± 4.9	0.009
HbA1c%	7.5 ± 2.4	7.6 ± 2.4	7.5 ± 2.4	0.14
Serum cholesterol (mmol/L)	4.9 ± 1.6	4.8 ± 1.8	4.9 ± 1.5	0.20
Serum triglyceride (mmol/L)	1.8 ± 1.2	1.7 ± 1.4	1.8 ± 1.1	0.002
Serum HDL (mmol/L)	1.0 ± 0.3	1.2 ± 0.3	0.9 ± 0.3	< 0.001
Serum LDL (mmol/L)	3.1 ± 1.1	2.9 ± 1.1	3.1 ± 1.1	< 0.001
Systolic blood pressure (mmHg)	156.8 ± 30.4	152.2 ± 30.3	157.9 ± 30.4	< 0.001
Diastolic blood pressure (mmHg)	90.6 ± 19.3	82.9 ± 18.0	92.4 ± 19.2	< 0.001
Body mass index on admission (kg/m^2^)	27.7 ± 5.1	29.7 ± 6.7	27.2 ± 4.5	< 0.001
Antiplatelets at discharge	6,097 (83.5)	1,096 (78.0)	5,001 (84.8)	< 0.001
Anticoagulants at discharge	543 (7.4)	147 (10.5)	396 (6.7)	< 0.001
Anti-hypertensive at discharge	4,707 (64.5)	920 (65.4)	3,787 (64.3)	0.41
Antidiabetics at discharge	2,843 (38.9)	601 (42.7)	2,242 (38.0)	< 0.001
Statin at discharge	6,191 (84.8)	1,141 (81.2)	5,050 (85.7)	< 0.001

HDL, high-density lipoprotein; LDL, low-density lipoprotein; RBS, random blood sugar.

### Mode of transportation to the hospital, course in hospital, and acute stroke treatment

Most patients arrived at the hospital via activation of the ambulance service (67.5%). Use of the ambulance service was, however, significantly lower among women (women: 64.3% vs. men: 68.2%; *p* = 0.001). Women were more likely to present to the hospital later after the onset of symptoms. Nearly 32% of patients presented to the hospital within 4.5 h of onset (women: 29% vs. men: 32.8%; *p* = 0.01). Significantly more women presented to the hospital more than 24 h after the onset of symptoms (women: 36.6% vs. men: 33.2%; *p* = 0.01). Women were also more likely to report that they were unsure about the time of onset (women: 8.3% vs. men: 6.9%; *p* < 0.01). Female patients had significantly lower systolic blood pressure (women: 152.2 ± 30.3 vs. men: 157.9 ± 30.4; *p* < 0.001) and diastolic blood pressure (women: 82.9 ± 16.7 vs. men: 92.4 ± 19.2; *p* < 0.001) at admission. Severe stroke (NIHSS > 10) was significantly more common among women than among men (19.9% vs. 14.5%; *p* < 0.001). Intravenous thrombolysis with rt-PA was, however, less likely to be offered to women compared to men (women: 9.8% vs. men: 12.1%; *p* = 0.02). Door-to-needle time among patients offered this treatment was significantly longer for women compared to men (women: 65.9 ± 35.5 min vs. men: 58.9 ± 34.4 min; *p* = 0.03). Thrombectomy was carried out in 4.3% of patients, and there was no difference in rate between women and men. Decompressive hemicraniectomy was carried out in a small number of patients (1.3%), and was significantly less common in women (women: 1.5% vs. men: 0.8%; *p* = 0.04). There was no difference in the rate of post-rt-PA cerebral hemorrhage (women: 5.0% vs. men: 6.6%; *p* = 0.49). The frequencies of various reasons for not offering rt-PA are shown in Table 2; rates of post-rt-PA symptomatic hemorrhage are also shown in Table 2.

**Table 2:** Comparison of admission and in-hospital parameters between female and male ischemic stroke patients.

**Variable**	**Overall (*n* = 7,300)**	**Women (*n* = 1,406, 19.3%)**	**Men (*n* = 5,894, 80.7%)**	***P*-value**
**Mode of arrival**				
Ambulance service	4,926 (67.5)	904 (64.3)	4,022 (68.2)	< 0.001
Non-medical transport	1,952 (26.7)	404 (28.7)	1,548 (26.3)	
Already in hospital	152 (2.1)	52 (3.7)	100 (1.7)	
From another hospital	270 (3.7)	46 (3.3)	224 (3.8)	
NIHSS on admission	5.5 ± 5.9	6.1 ± 6.6	5.3 ± 5.7	< 0.001
**NIHSS severity**				
Mild (NIHHS 0–4)	4,452 (61.0)	816 (58.0)	3,636 (61.7)	< 0.001
Moderate (NIHSS 5–10)	1,715 (23.5)	310 (22.0)	1,405 (23.8)	
Severe (NIHSS >10)	1,133 (15.5)	280 (19.9)	853 (14.5)	
**Arrival time from onset**				
< 4.5 h	2,340 (32.1)	409 (29.1)	1,931 (32.8)	0.011
< 24 h	1,703 (23.3)	319 (22.7)	1,384 (23.5)	
>24 h	2,471 (33.8)	515 (36.6)	1,956 (33.2)	
Wake-up stroke	265 (3.6)	46 (3.3)	219 (3.7)	
Unsure of onset timing	521 (7.1)	117 (8.3)	404 (6.9)	
IV thrombolysis	849 (11.6)	138 (9.8)	711 (12.1)	< 0.02
Thrombectomy	312 (4.3)	62 (4.4)	250 (4.2)	0.78
**Reasons for no thrombolysis**				
Out of window	4,649 (72.1)	920 (72.6)	3,729 (71.9)	0.02
Low NIHSS/improved	978 (15.2)	166 (13.1)	812 (15.7)	
High NIHSS/established or large infarct	331 (5.1)	61 (4.8)	270 (5.2)	
Wake-up stroke/unsure of onset/missed/ late/declined	130 (2.0)	30 (2.4)	100 (1.9)	
Contraindications/other reasons	363 (5.6)	90 (7.1)	273 (5.3)	
Post-thrombolysis bleed	54 (6.3)	7 (5.0)	47 (6.6)	0.49
Length of stay	5.7 ± 6.9	6.4 ± 7.6	5.5 ± 6.8	< 0.001
Complications during admission	599 (8.2)	165 (11.7)	434 (7.4)	< 0.001
Aspiration pneumonia	292 (4.0)	60 (4.3)	232 (3.9)	0.57
Urosepsis	187 (2.6)	68 (4.8)	119 (2.0)	< 0.001
Bedsores	36 (0.5)	16 (1.1)	20 (0.3)	< 0.001
Sepsis	209 (2.9)	62 (4.4)	147 (2.5)	< 0.001
Deep venous thrombosis	6 (0.1)	1 (0.1)	5 (0.1)	0.87
**TOAST classification**				
Small vessel disease	3,460 (47.4)	621 (44.2)	2,839 (48.2)	< 0.001
Large vessel disease	1,541 (21.1)	261 (18.6)	1,280 (21.7)	
Cardioembolism	1,412 (19.3)	352 (25.0)	1,060 (18.0)	
Stroke of other determined etiology	553 (7.6)	100 (7.1)	453 (7.7)	
Stroke of undetermined etiology	334 (4.6)	72 (5.1)	262 (4.4)	

NIHSS, National Institute of Health Stroke Scale; IV, intravenous; TOAST, trial of Org 10,172 in acute stroke treatment.

Medical complications were significantly more common in women compared to men (women: 11.7% vs. men: 7.4%; *p* < 0.001), as shown in Table 2. Although the rate of aspiration pneumonia was similar in both sexes, other complications (including urosepsis, bedsores, and systemic sepsis) were significantly more common among female patients. Women also tended to have a significantly more prolonged length of stay in the hospital (women: 6.4 ± 7.6 vs. men: 5.5 ± 6.8; *p* < 0.001). Fewer female patients were taking antiplatelet medications at discharge (women: 78% vs. men: 84.8%; *p* < 0.001), but more female patients were discharged on anticoagulant medications (women: 10.5% vs. men: 6.7%; *p* < 0.001). The number of patients discharged on anti-hypertensive medications was similar for both sexes (women: 65.4% vs. men: 64.3%; *p* = 0.41). More female patients were discharged on antidiabetic medications (women: 42.7% vs. men: 38.0%; *p* < 0.001). The use of statins, however, was less frequent in women (women: 81.2% vs. men: 85.7%; *p* < 0.001). Significantly fewer female patients received long-term rehabilitation care (women: 16.8% vs. men: 23.5%; *p* < 0.001). The proportion of patients falling into each final TOAST classification, representing the etiology of stroke diagnosis, is shown in Table 2. There were significantly more women with a diagnosis of cardioembolic stroke (women: 25% vs. men: 18%; *p* < 0.001).

### Short-term outcomes at 90-day follow-up and MACEs at 1-year follow-up in ischemic stroke patients

**Figure 2:**
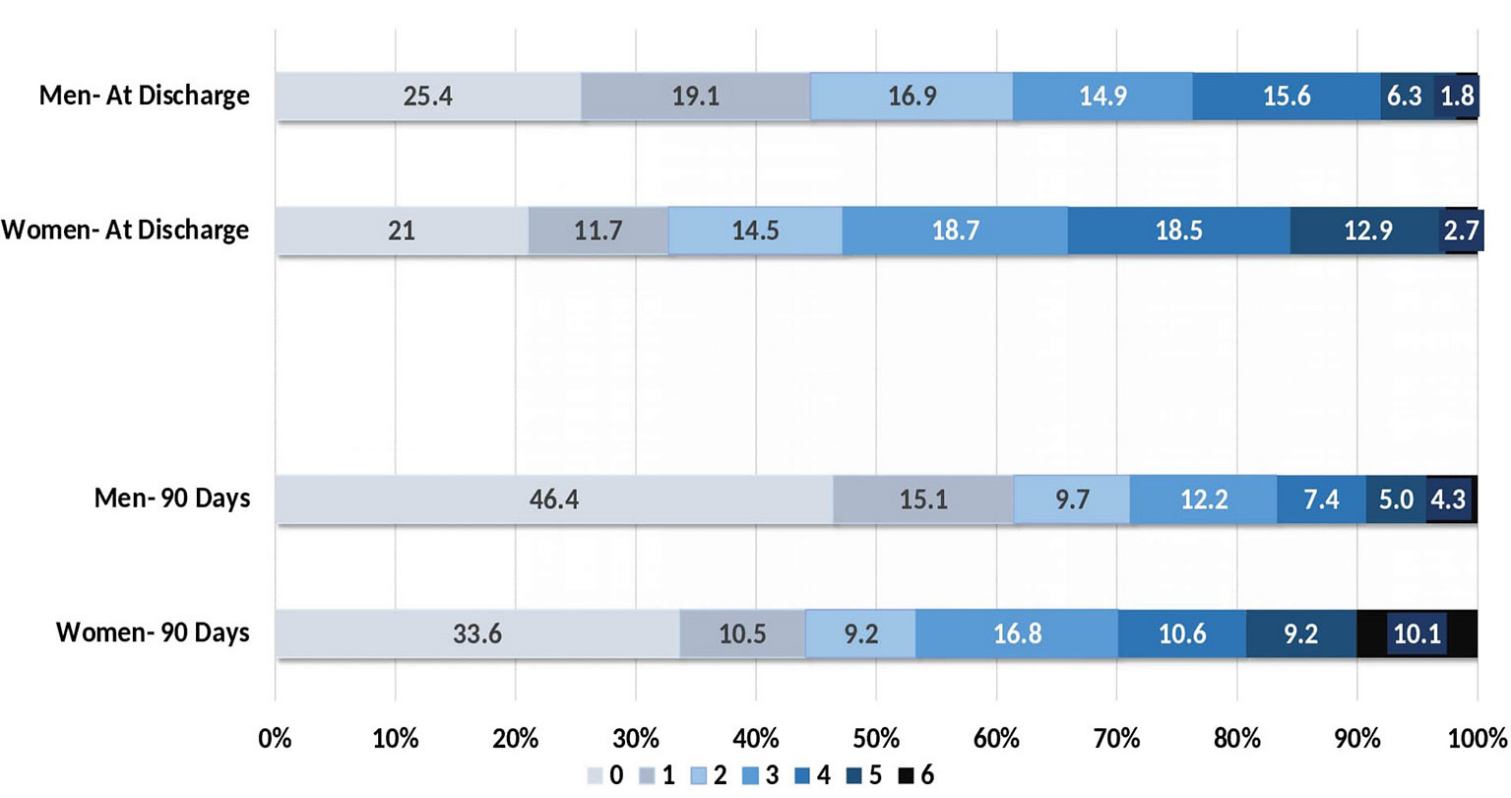
Modified Rankin Scale scores at discharge and at 90 days for women and men.

Short-term outcomes were measured in the form of mRS scores at discharge and at 90 days. As shown in Figure 2, an mRS score of 0–2, implying a good outcome, was observed in 47.2% of female patients and 61.3% of male patients at discharge (*p* < 0.001). At 90-day follow-up, there were still significantly fewer female patients with a good outcome (women: 53.3% vs. men: 71.2%; *p* < 0.001). Among female patients, prognosis was related to age at the time of presentation. A poor prognosis at 90 days was most likely among female patients over the age of 65 (women: 62.9% vs. men: 29.9%; *p* = 0.001; Table 3). Additionally, significantly more deaths occurred among women, both during the hospital stay (women: 2.7% vs. men: 1.8%; *p* = 0.02) and during the 90 days following discharge (women: 10.1% vs. men: 4.3%; *p* < 0.001), as shown in Table 3.

**Table 3:** Differences between female and male ischemic stroke patients in terms of short-term and 1-year outcomes.

**Outcome measure**	**Overall (*n* = 7,300)**	**Women (*n* = 1,406, 19.3%)**	**Men (*n* = 5,894, 80.7%)**	***P*-value**
**mRS score at 90 days**				
0	2,470 (43.8)	382 (33.6)	2,088 (46.4)	< 0.001
1	799 (14.2)	119 (10.5)	680 (15.1)	
2	541 (9.6)	104 (9.2)	437 (9.7)	
3	739 (13.1)	191 (16.8)	548 (12.2)	
4	451 (8.0)	120 (10.6)	331 (7.4)	
5	328 (5.8)	105 (9.2)	223 (5.0)	
6	308 (5.5)	115 (10.1)	193 (4.3)	
**mRS category at discharge**				
Good (mRS 0–2)	4,279 (58.6)	664 (47.2)	3,615 (61.3)	< 0.001
Poor (mRS 3–6)	3,021 (41.4)	742 (52.8)	2,279 (38.7)	
**mRS category at 90 days (*****n*** = **5,636, 77.2%)**				
Good (mRS 0–2)	3,810 (67.6)	605 (53.3)	3,205 (71.2)	< 0.001
Poor (mRS 3–6)	1,826 (32.4)	531 (46.7)	1,295 (28.8)	
**Poor outcome (mRS 3–6) at 90 days (*****n*** = **1,826)**				
Age < 46 years	312 (17.1)	42 (7.9)	270 (20.8)	< 0.001
Age 46–55 years	382 (20.9)	45 (8.5)	337 (26.0)	
Age 56–65 years	450 (24.6)	110 (20.7)	340 (26.3)	
Age >65 years	682 (37.3)	334 (62.9)	348 (26.9)	
Mortality rate at discharge	144 (2.0)	38 (2.7)	106 (1.8)	0.03
Mortality rate at 90 days	308 (5.5)	115 (10.1)	193 (4.3)	< 0.001
**1-year outcomes**				
Recurrent stroke (ischemic or hemorrhagic)	132 (2.3)	28 (2.4)	104 (2.3)	0.85
Post-stroke MI (fatal or non-fatal)	44 (0.8)	9 (0.8)	35 (0.8)	0.99
All-cause mortality	372 (6.5)	139 (11.7)	233 (5.1)	< 0.001
Post-stroke cardiac arrest	94 (1.7)	37 (3.2)	57 (1.3)	< 0.001
Post-stroke cardiac revascularization (CABG or PCI)	44 (0.8)	7 (0.6)	37 (0.8)	0.46
Major cardiac adverse event (MACE)	561 (7.7)	177 (12.6)	384 (6.5)	< 0.001
**Total MACEs over 5 years**				
0 events	6,739 (92.3)	1,229 (87.4)	5,510 (93.5)	< 0.001
1 event	439 (6.0)	134 (9.5)	305 (5.2)	
2 events	119 (1.6)	43 (3.1)	76 (1.3)	
3 events	3 (0.1)	0	3 (0.1)	

mRS, Modified Rankin Scale; MI, myocardial infarction; CABG, coronary artery bypass graft; PCI, percutaneous coronary intervention; MACE, major adverse cardiovascular event.

For assessment of outcomes at 1-year follow-up, we analyzed the occurrence of MACEs in the two groups. As shown in Table 3, significantly more female patients experienced at least one MACE during the year following discharge from the hospital (women: 12.6% vs. men: 6.5%; *p* < 0.001). The rate of recurrent stroke was similar among both sexes, but women were more likely than men to undergo cardiac arrest and to die. Women were also more likely to experience recurrent events compared to men. The higher incidence of events during follow-up may be related to the older age, higher incidence of vascular risk factors, and more frequent co-morbidities evident in women.

### Multivariate analysis

We performed a multivariate logistic regression analysis to identify factors associated with women in comparison to men among ischemic stroke patients.

The rate of obesity was 2.5 times greater in female patients compared to male patients (adjusted OR: 2.5, 95% CI: 2.1–2.9). Similarly, a TOAST classification of ischemic stroke of unknown etiology was observed 1.5 times more frequently in female patients compared to male patients (adjusted OR: 1.4, 95% CI: 1.02–2.0). The rates of other diagnoses (small vessel disease, large vessel disease, cardioembolism, and stroke of other determined etiology) did not differ significantly between the sexes.

**Figure 3:**
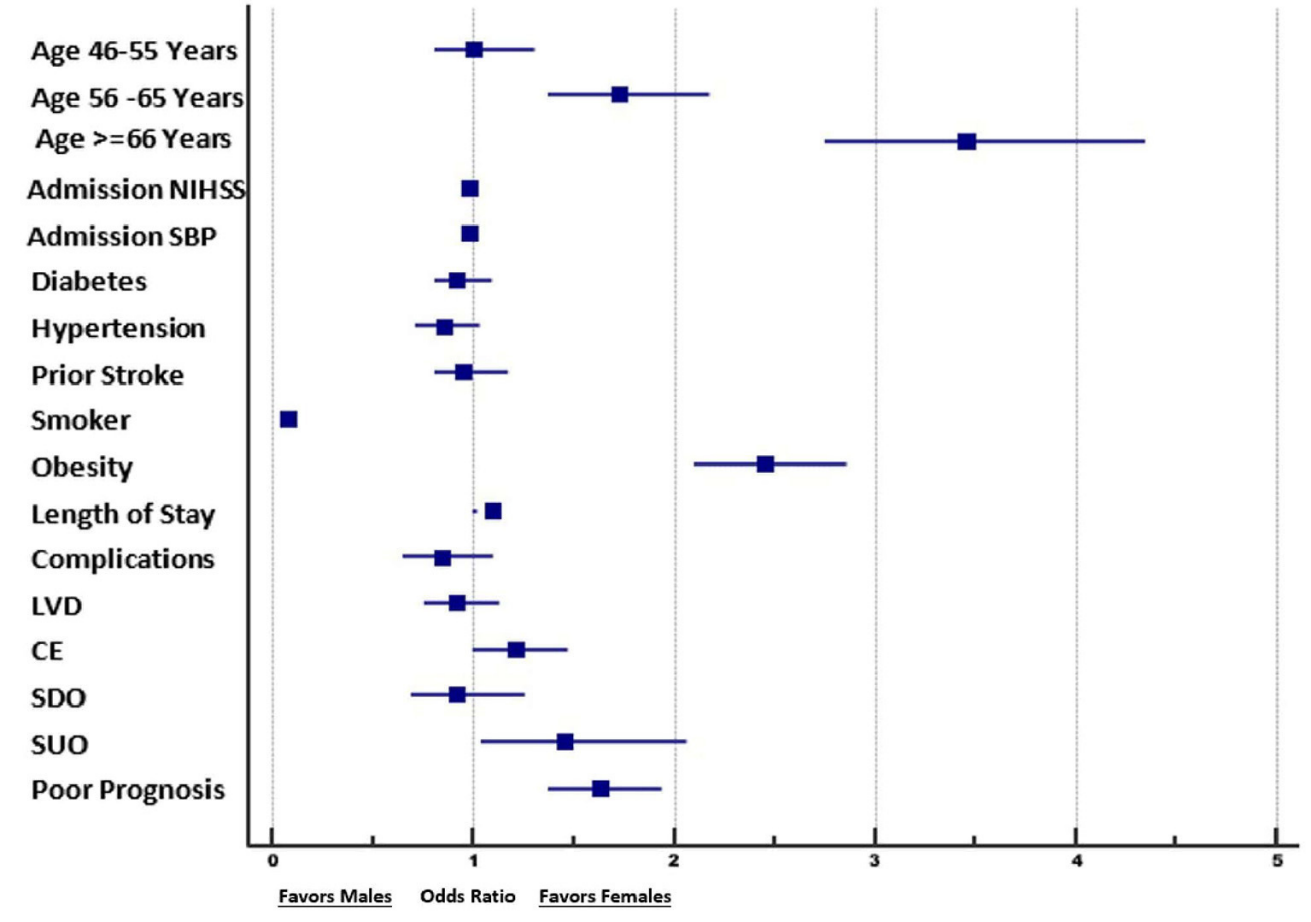
Forest plot of the results of the logistic regression analysis, showing the factors associated with female patients vs. male patients. NIHSS, National Institute of Health Stroke Scale; SBP, systolic blood pressure; LVD, large vessel disease; CE, cardioembolic stroke; SDO, stroke of determined origin; SUO, stroke of undetermined origin.

After adjusting for associated factors in the multivariate logistic regression, including age, NIHSS on admission, DM on admission, HTN on admission, prior stroke history, active smoker status, obesity, LOS, complications during admission, and TOAST classification, the likelihood of a poor outcome (mRS score 3–6) at 90 days was found to be greater among women than among men (adjusted OR: 1.61, 95% CI: 1.30–1.92). The details are shown in Tables 4, 5 and in Figure 3.

**Table 4:** Multivariate binomial logistic regression of factors associated with sex among ischemic stroke patients.

**Variable**	**Adjusted odds ratio**	**95.0% CI**		***P*-value**
		**Lower**	**Upper**	
**Age**				
< 46 years	1.00	–	–	< 0.001
46–55 years	0.99	0.79	1.26	0.96
56–65 years	1.72	1.37	2.17	< 0.001
>65 years	3.43	2.73	4.31	< 0.001
Systolic blood pressure (mmHg)	0.99	0.993	0.998	0.002
Diabetes	0.94	0.80	1.09	0.42
Hypertension	0.87	0.72	1.04	0.13
Prior stroke	0.95	0.77	1.18	0.65
Smoking status	0.08	0.06	0.11	< 0.001
Obesity (kg/m^2^)	2.46	2.10	2.87	< 0.001
Length of stay	1.00	0.99	1.01	0.69
Poor outcome at 90 days (mRS score 3–6)	1.49	1.27	1.76	< 0.001
**TOAST classification**				
Small vessel disease		–	–	0.03
Large vessel disease	0.88	0.72	1.08	0.22
Cardioembolism	1.15	0.95	1.39	0.14
Stroke of other determined etiology	0.90	0.67	1.21	0.49
Stroke of undetermined etiology	1.41	0.99	2.00	0.05

Variables entered into the model: age category; SBP on admission; diabetes status on admission; hypertension status on admission; prior stroke history; active smoker status; obesity (BMI ≥ 30); length of hospital stay; poor prognosis at 90 days; and TOAST classification of ischemic stroke.

**Table 5:** Multivariate binomial logistic regression analysis of factors associated with poor outcome (mRS score 3–6) at 90 days in ischemic stroke patients.

**Variable**	**Adjusted odds ratio**	**95.0% CI**		***P*-value**
		**Lower**	**Upper**	
Age (years)	1.05	1.04	1.05	< 0.001
Female sex	1.7	1.39	1.99	< 0.001
NIHSS on admission	1.2	1.18	1.21	< 0.001
Systolic blood pressure (mmHg)	1.00	1.000	1.005	0.09
Diabetes	1.28	1.09	1.49	0.002
Hypertension	1.22	1.02	1.47	0.03
Prior stroke	1.62	1.32	1.99	< 0.001
Smoking status	0.96	0.81	1.14	0.65
Obesity (kg/m^2^)	0.92	0.78	1.09	0.34
Length of hospital stay	1.14	1.12	1.16	< 0.001
**TOAST classification**				
Small vessel disease				0.001
Large vessel disease	1.22	1.02	1.48	0.03
Cardioembolism	1.01	0.83	1.24	0.91
Stroke of other determined etiology	0.68	0.49	0.92	0.01
Stroke of undetermined etiology	1.44	1.01	2.05	0.04

NIHSS, National Institute of Health Stroke Scale.

Variables entered into the model: age (years); sex (female = 1); NIHSS on admission; SBP on admission; diabetes status on admission; hypertension status on admission; prior stroke history; active smoker status; obesity (BMI ≥30); length of hospital stay; and TOAST classification of ischemic stroke.

## Discussion

This is the largest study using prospectively collected data on the role of sex in terms of evaluation, management and prognosis, and long-term outcomes for ischemic stroke patients in the Middle East. The patients represented multiple ethnicities, but were predominantly Arabic or from Southeast Asia. The significantly higher average age among women reflected the disproportionately high number of young male expatriate workers in Qatar compared to women, as we have previously reported (22–26). We have previously shown that stroke occurs at a younger age among expatriate subjects and that this is likely to be related to undiagnosed or poorly controlled risk factors. We have also previously shown that a higher incidence of lacunar stroke occurs among patients with a low NIHSS score, which is also related to poorly controlled hypertension and diabetes (22–26).

The present research revealed that, despite presenting with more severe stroke, women reported to the hospital later, underutilized the ambulance service, and were significantly less likely to receive intravenous thrombolysis treatment. In addition, the time to treatment (door-to-needle time) was also significantly delayed in women compared to men. The significantly higher rate of medical complications among women while in hospital can be explained by the older age of this group, the higher incidence of diabetes and obesity, and the likelihood of more severe stroke at presentation. These are all likely contributors to lower rates of recovery at discharge and at 90-day follow-up. Despite greater severity of stroke, fewer women than men were admitted for rehabilitation. Our research also showed that the incidence of MACEs during the 1-year follow-up period was significantly higher among women. This may in part be explained by their older age and the more frequent presence of diabetes. However, significantly fewer women were receiving antithrombotic, antidiabetic, or statin medications at the time of discharge, even though the incidence of diabetes was higher in women. These are important observations and require further analysis in the context of previously published literature on women and stroke. Special attention is required for women in order to improve awareness of the early signs of stroke, achieve rapid admission to the hospital, increase provision of reperfusion treatment, improve provision of care to prevent acute stroke-associated medical complications, and provide aggressive post-stroke care to improve prognosis and decrease the incidence of MACEs during follow-up.

Stroke is more common in women compared to men throughout life (1), and is especially prominent as a cause of death in the elderly (29). Elderly women are more likely to live alone and to have a higher degree of disability, making it more difficult for them to reach the hospital in good time (29). Treatable risk factors, including hypertension, diabetes, and atrial fibrillation, are also more common in the elderly and (as was evident from our study) may be undertreated. In our study, 50% of female patients with atrial fibrillation were not taking anticoagulation medication, and this likely accounted for the higher rate of cardioembolic stroke. We also observed that the use of statins was significantly lower in female patients at the time of discharge, despite this group having a significantly higher incidence of diabetes. Undertreatment of diabetes (30) and lower rates of statin use (31) in women have previously been noted. The use of antithrombotic medications was also significantly lower in women, an observation that has previously been noted (21). Our study shows that there focused attention needs to be placed on better management of vascular risk factors before and after stroke. Given the high rates of MACEs observed in women, this is especially critical following discharge after admission for acute stroke.

Women are significantly delayed in their arrival at the hospital and are less likely to receive rt-PA (32). In our study, fewer female patients presented within the 4.5-h time window, and women were less likely to use the ambulance service, which contribution to delay in their evaluation. The longer door-to-needle time observed in our study may also be a contributing factor in the fact that fewer female patients make a good recovery (33). Furthermore, women experience significantly higher rates of medical complications during hospitalization. Medical complications, especially aspiration pneumonia and sepsis, are important causes of increased mortality in stroke patients (34) and have been underreported in recent reviews on stroke in women (1, 2). Older age at presentation and greater neurological deficits at presentation are also important risk factors for medical complications in acute stroke patients. The combination of these factors likely contributed to the longer hospital stays and higher rate of mortality observed in women in our study. In our study, these higher rates of poor recovery and mortality in women persisted even after correction for the age difference in our multivariate analysis.

Our study showed a significantly lower rate of good recovery in women compared to men. Multiple factors likely contributed to this difference, as discussed above in detail. Women had a 50% lower rate of recovery even after correction for age, risk factors, and occurrence of complications in the hospital. However, a meta-analysis published recently has suggested that baseline differences in age, vascular risk factors, and stroke etiology can explain most of the sex-related difference in mortality (2, 35). In our population, the higher door-to-needle time for women may also be related in part to local social and ethnic practices. Traditionally, treatment-related decisions are made by male members of the family. It is not uncommon to wait for a male family member (for example, the patient's husband or brother) to arrive at the hospital before treatment can be initiated.

Few studies have been conducted on the long-term risk of MACE in women (1–4). We have previously shown that depression is more common in women following a stroke in our population (28), and depression has been found to be associated with slower post-stroke recovery (28). There are additional significant factors that may also contribute to the fact that fewer women make a good recovery and that MACEs occur at higher rates in women. Women are more likely to experience mobility issues (36), cannot tolerate pain well (1, 2), and are more likely than men to be single or widowed (1, 2). In a recent study conducted in London, in which patients were followed for more than 10 years following acute stroke, women were older and had significantly poorer outcomes in terms of activities of daily living (37). Despite their older age at presentation, women were less likely to experience recurrent strokes and had a lower rate of death during follow-up (37). In our study, we observed a higher rate of MACE in women during 1-year post-stroke follow-up. This may in part be related to lower rates of preventive treatment at the time of discharge. Despite the higher incidence of diabetes in women, we noticed that the use of antidiabetic medications and statins was lower in women than in men.

Our study shows that attention to some of the factors identified may lead to better outcomes in female patients. Vascular risk factors, especially diabetes, hypertension, obesity, and atrial fibrillation, were more common in women, and yet at the time of discharge, women were less likely to be offered statins and antithrombotic medications. Women were also less likely to receive rehabilitation treatment and had higher all-cause mortality during follow-up. Attention to strategies that may improve early admission to hospital, higher rates of thrombolysis, reduction in medical complications, and improved management of vascular risk factors may contribute to improving the care of female patients who suffer from acute stroke. The results are similar to those of a recent report from China on 9,038 patients, among whom stroke occurred at an older age and the outcomes were worse in women (38). The unfavorable outcome remained after correction for age, risk factors, and onset-to-door time (38). Lower acceleration of biological age for ischemic stroke in women may in part explain the older age at presentation (39).

There are limitations to our study. This was a single-center study and therefore may not reflect stroke practices across the entire Middle East. The data were collected prospectively for entry into the Qatar stroke database, but the analysis was retrospective and therefore did not have prespecified objectives. The male patients included were younger and the majority were expatriates, while the women were older and a larger proportion represented the Qatari and Arab population. The older age is likely responsible for the higher percentage of women who made a slower recovery, but does not explain why fewer patients were offered thrombolysis acutely or the lower rates of treatment with preventative therapies. For analysis of MACE incidence, we used electronic medical records. We may have missed clinical events occurring in patients who did not go to the hospital or who may have received treatment outside the country. Finally, the population of Qatar is skewed, with a larger proportion of younger expatriate men. This may account for the higher percentage of women in the older segment of the patient population.

In summary, we present a large study from the Middle East on sex differences in the management of acute stroke. Our study shows that, compared to men, women are significantly older at presentation, experience longer delays in coming to the hospital, and are less likely to receive thrombolysis. We also showed that women are more likely to suffer in-hospital complications and make a slower recovery. Despite higher rates of disease burden and vascular risk factors, fewer women are discharged on antithrombotic, statin, or antidiabetic medications.

## Data availability statement

The original contributions presented in the study are included in the article/supplementary material, further inquiries can be directed to the corresponding author.

## Ethics statement

The study was approved by the Institutional Review Board, Hamad Medical Corporation at the Medical Research Center (MRC-01-20-1135) and the Institutional Review Board of Weill Cornell Medicine-Qatar (1932095-1/22-00016). Written informed consent for participation was not required for this study in accordance with the national legislation and the institutional requirements.

## Author contributions

HN, NA, and AS: conceptualization, design, and drafting of the manuscript. HN, MA, BK, AN, NA, and AS: acquisition, analysis, and interpretation of data and technical and administrative support. SK, SA, and AS: critical review. RS, ST, and NA: statistical analysis. All authors contributed to the article and approved the submitted version.
